# Gleditsia Saponin C Induces A549 Cell Apoptosis via Caspase-Dependent Cascade and Suppresses Tumor Growth on Xenografts Tumor Animal Model

**DOI:** 10.3389/fphar.2017.00988

**Published:** 2018-01-15

**Authors:** Ye Cheng, Weidong He, Yongming He

**Affiliations:** ^1^Department of Integrated Traditional Chinese and Western Medicine, Jiangsu Cancer Hospital, Jiangsu Institute of Cancer Research and Nanjing Medical University Affiliated Cancer Hospital, Nanjing, China; ^2^Department of Orthopedics, Affiliated Hospital of Integrated Traditional Chinese and Western Medicine, Nanjing University of Chinese Medicine and Jiangsu Province Academy of Traditional Chinese Medicine, Nanjing, China

**Keywords:** natural product, gleditsia saponin C, apoptosis, lung cancer, drug development

## Abstract

Saponins are natural compounds and possess the most promising anti-cancer function. Here, a saponin gleditsia saponin C (GSC), extracted from gleditsiae fructus abnormalis, could induce apoptosis of lung tumor cell line A549 via caspase dependent cascade and this effect could be prevented by the caspase inhibitors. In addition, GSC induced cell death companied with an increase ratio of Bax:Bcl-2 and inhibition of ERK and Akt signaling pathways. Meanwhile, GSC suppressed TNFα inducing NF-κB activation and increased the susceptibility of lung cancer cell to TNFα induced apoptosis. Furthermore, on mouse xenograft model, GSC significantly suppressed tumor growth and induced cancer cell apoptosis, which validated the anti-tumor effect of GSC. Based on these results, GSC might be a promising drug candidate of anti-lung cancer for its potential clinical applications.

## Introduction

Lung cancer is characterized by un-controlled cell growth in lung tissues. The tumor growth can also spread beyond the lung by metastasis into nearby tissue or other parts of the body. In 2012, lung cancer occurred in 1.8 million people and resulted in 1.6 million deaths, which becomes the most common cause of cancer-related death in men and second most common in women after breast cancer. More astonishingly, only 17.4% of people in the United States diagnosed with lung cancer survive 5 years after the diagnosis, while outcomes on average are worse in the developing world (Stewart and Wild, 2014).

Small-cell lung cancer and NSCLC are two main types of lung cancer. NSCLC is responsible for more than 80% of total lung cancer cases (Ross and Mann, 2017). Though chemotherapy, radiation therapy, immunotherapy, and surgery have been applied for treatment, many cancer patients still show resistance to current therapies. Therefore, it is urgent to develop new drugs to maximize the antitumor potency and minimize the side effects of commonly prescribed chemotherapy drugs.

**FIGURE 1:**
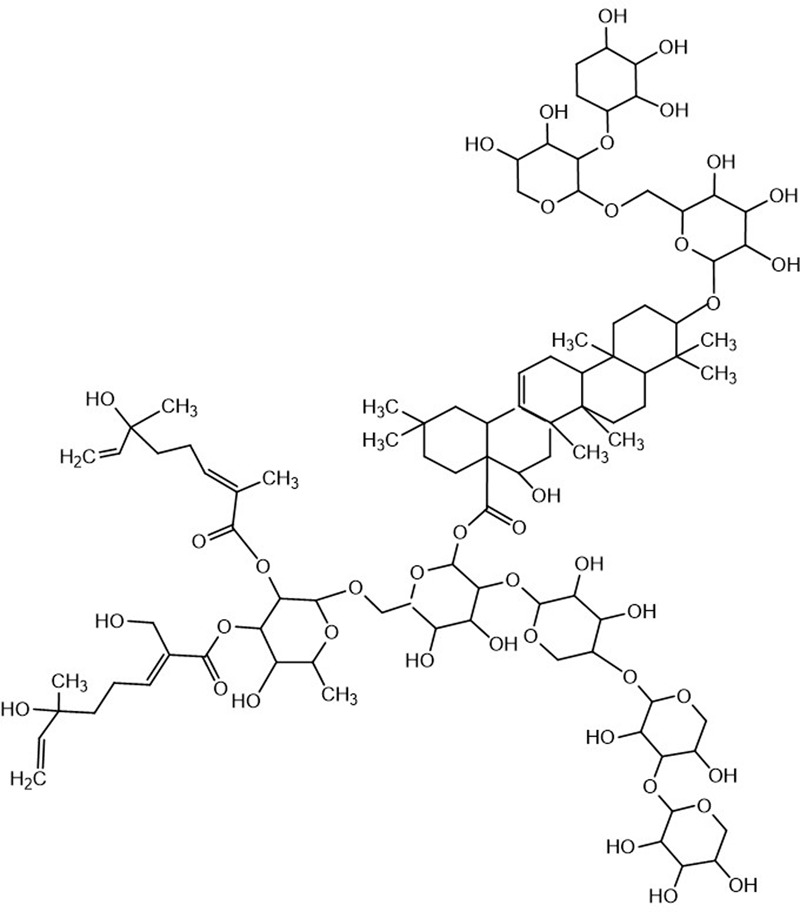
Chemical structure of GSC.

Traditional Chinese herbal remedies have become an important resource for developing new anticancer drugs and novel chemotherapy complements to improve the competence of cancer chemotherapy or to relieve chemotherapy side effects. Despite the healing mechanisms are not fully discovered, some drugs and therapies have helped patients battle cancers with fewer side effects and higher potency than other treatments. Chemicals extracted from herbs, which include saponin compounds, have been shown to exhibit significant antitumor functions. Gleditsia saponin C (GSC, as shown in **Figure 1**) is a saponin isolated from gleditsiae fructus abnormalis (the dried infertile fruits of *Gleditsia sinensis* Lam., which is widely prescribed to eliminate phlegm, regain consciousness, disperse accumulation of evils and carbuncle in TCM, and to treat lung cancer in clinic nowadays (Lao et al., 2012). GSC has exerted profound cytotoxicity on cancer cell model. However, the concrete action mechanism is unknown.

Apoptosis induction is a critical stage for compounds exerting anti-cancer function. In general, apoptosis include signaling pathways via death receptor (extrinsic) and mitochondria (intrinsic). By coupled with ligand such as TNFα, extrinsic pathway initiates downstream caspases, such as caspase-8, -3, -6, -7, in series caspase cascade, which subsequently cleaves a broad spectrum of target substrates and leads to cell apoptosis. The intrinsic pathway includes mitochondrial permeability transition changes as well as mitochondrial membrane potential alterations. Afterward, apoptogenic factors, e.g., AIF and cytochrome c are released from the mitochondria into the cytosol. Apoptosis is mediated by a number of molecules that either inhibit (including Bcl-xl, Bcl2, and the IAP family of proteins) or induce (such as Bak, Bad, and caspases) cell death (Aouacheria et al., 2017). Defective apoptosis is a key factor in tumorigenesis as well as in treatment resistance. Therefore, apoptotic pathways are hot targets of cancer therapies and related drug development.

Therefore, we investigated the effects of GSC on cell viability as well as apoptosis induction in human lung cancer cell lines. The MAPK cascade, caspase activation, and NF-κB pathway are all found to be involved in apoptosis induction mechanisms of GSC. Furthermore, GSC could increase the susceptibility of cancer cells to TNFα-triggered cell death via inhibiting NF-κB activity. In addition, anti-apoptosis effect of GSC was validated on xenograft tumor animal model, which further proved its potentiality of anti-lung cancer drug development.

## Materials and Methods

### Reagents

Gleditsia saponin C (purity > 95%) was purchased from Sichuan Weikeqi Biological Technology, Co., Ltd. (Chengdu, China), and was dissolved in dimethyl sulfoxide (DMSO) for cell culture; a final culture concentration of DMSO was ≤0.1%. U0126 (Selleck, China), LY294002 (Selleck, China), and AZD6244 (Selleck, China) were freshly dissolved in DMSO and IGF-1 (R&D, Minnesota) were freshly dissolved in culture medium each time before use. LPS (*Escherichia coli* 055:B5) was purchased from Sigma Chemical, Co. (St. Louis, MO, United States). Annexin V and PI were purchased from Molecular Probes (Eugene, OR, United States). All of the reagents for cell cultures were purchased from Thermo Fisher Scientific (Invitrogen, Carlsbad, CA, United States).

### Cell Cultures

A549, H1299 cell lines, and BEAS-2B cells were purchased from the American Type Culture Collection (ATCC, Philadelphia, PA, United States) and were grown in RPMI 1640 supplemented with 10% (v/v) fetal bovine serum (FBS) and 1% penicillin-streptomycin. All cells were cultured in a humidified 5% CO_2_ incubator at 37°C.

### Flow Cytometry

Cells were treated with series dilution of GSC solution in the presence or absence of either TNFα (20 ng/mL), or U0126 (2 μM), or LY294002 (5 μM). After 24 h treatment, the ratio of cell apoptosis was determined by flow cytometry analysis. In details, after incubation of different treatment for 24 h, the cells were detached, washed with phosphate-buffered saline (PBS), and centrifuged at 1,000 rpm for 5 min. FITC-labeled Annexin V and PI (5 μL each) were added to 500 μL of the cell suspension, mixed, incubated at room temperature for 5–15 min in the dark, and then the cells were analyzed using flow cytometer (FACSCalibur, BD Instruments, Inc., United States).

### Cell Proliferation Assay

The effects of GSC on cell proliferation were evaluated by MTT assay. Cells were seeded into a 96-well plate at a density of 5,000 cells per well. After 24 h, 0–40 μM GSC was added to the medium. The cells were incubated at 37°C for 24 h, and then the cell viability was determined by the colorimetric MTT [3-(4, 5-dimethylthiazol-2-yl)-2, 5-diphenyl-2H-tetrazolium bromide] assay at wave length 570 nm by using a microplate reader (Bio-Rad, Hercules, CA, United States). The cell viability was calculated according to the formula: Cell viability (%) = average A570 nm of treated group/average A570 nm of control group × 100%.

### Western Blot Analysis

Total cell lysates were extracted by using radioimmunoprecipitation assay (RIPA) buffer (Solarbio, Beijing, China). Nucleus proteins were extracted by using nucleus protein extraction kit (Beyotime, Shanghai, China). Lysates were collected and centrifuged at 12,000 rpm. Loading buffer was added to the supernatant of samples and the proteins were denatured at 100°C for 5 min. Proteins were separated by 12% sodium dodecyl sulfate–polyacrylamide gel electrophoresis (SDS–PAGE) and then transferred to PVDF membrane. The membranes were blocked with 5% non-fatted milk, washed four times with Tris-buffered saline plus Tween (TBS-T, 15 min each time), and then incubated with the following primary antibodies: p-JNK, JNK, p-Akt (Ser 473), Akt, p-PI3K, PI3K, Fas, cleaved caspase-3, 7, 8, 9, p-IKK-β, IKK-β, p-IκBα, IκBα, NFκB p65, p-ERK, ERK, p-p38, p38, poly(ADP-ribose) polymerase (PARP), Bad, Bax, Bcl-xl, Bcl-2, Lamin B, GAPDH (Cell Signaling Technology, Beverly, MA, United States). After overnight incubation at 4°C, the membranes were washed four times with TBS-T and then incubated with HRP conjugated secondary antibodies according to each species for another 2 h at room temperature. The relative band density was determined by using the Bio-Rad Imaging System (Hercules, CA, United States) with an enhanced chemiluminescence (ECL) western blotting substrate kit (Tianmen, China).

### TUNEL Assays

Cells were treated with 0–20 μg/mL GSC for 24 h and then the TUNEL assay was performed by using the TdT-FragEL^TM^ DNA Fragmentation Detection Kit (Merck, Germany) according to the manufacture instruction.

### Animals

Male BALB/C nude mice (CAnN.Cg-*Foxn1*^nu^/CrlVr, 16–22 g and 4–5-week-old) were provided by Nanjing Medical University and housed under germ free conditions. Animal care and use were carried out strictly according to the ethical guidelines by Nanjing Medical University Animal Care and Use Committee, and the study protocol was approved by the local institution review board. The animals were randomly allocated and blinding process was employed strictly throughout animal studies.

### *In Vivo* Animal Tumor Model Experiment

A549 cells (5 × 10^5^ cells in 30 μL) were suspended in PBS and subcutaneously injected into the right axilla of the nude mice. Tumor volume was determined by measuring the two maximum perpendicular tumor diameters with calipers every other day. Drug treatment was initiated on the 7th day when the volume of tumor reached a volume of 50 mm^3^. The mice were treated with GSC (10, 20, 40 mg/kg, gavage) every 2 days for a total of 3 weeks. The mice of control group were treated with PBS. Tumor volume was calculated according to the formula, tumor volume = length × width^2^ × 0.52. Antitumor activities of drugs were evaluated by growth inhibition of tumor volume.

### H&E and TUNEL Assays

Tumor beard nude mice were euthanized on the 21st day and then tumor tissues were collected. The tumor tissues were fixed with 4% formaldehyde and then embedded in paraffin and chopped into sections (5 μm) for hematoxylin/eosin (H&E) staining. Apoptotic cells in tumor sections were visualized by the TUNEL technique according to the manufacturer’s instruction (Merck, Germany).

### Statistical Analysis

Statistical analysis was carried out using the SPSS software (version 13.0, SPSS, IBM, Corp., Armonk, NY, United States). Before ANOVA analysis, normal distribution test was carried out firstly. Multiple group comparisons were carried out by one-way ANOVA followed by a Bonferroni *post hoc* analysis. Paired data were analyzed by using two-tailed Student’s *t*-tests. Data were expressed as the mean ± SD. Statistically significant changes were classed as significant [^∗^] where *p* < 0.05, highly significant [^∗∗^] where *p* < 0.01.

## Results

### Effects of GSC on Growth of Lung Cancer Cell Lines

**FIGURE 2:**
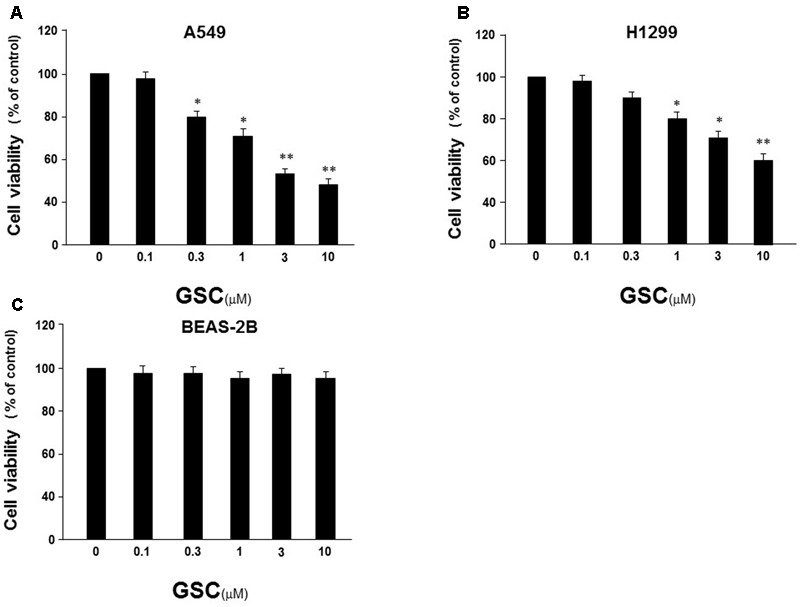
Effect of GSC on the viability of NSCLC cells. **(A)** A549 cells were treated with GSC at different concentrations (0, 0.1, 0.3, 1, 3, 10 μM) for 24 h, and the cell viability was determined by MTT assay. **(B)** The treatment of H1299 cells was same to **(A)** and the cell viability was determined by MTT assay. **(C)** The treatment of BEAS-2B cells was same to **(A)** and the cell viability was determined by MTT assay. The results were expressed as the mean ± SD of three independent experiments and each was performed in triplicate. ^∗^*p* < 0.05, ^∗∗^*p* < 0.01 (compared with that of non-GSC treated group).

Firstly, the effect of GSC on proliferation of A549 cells and H1299 cells was evaluated by MTT assay. These two cells were both treated with GSC for 24 h. Displayed in **Figures 2A,B**, the growth of A549 and H1299 were significantly inhibited with dose-dependent manners. Cell viability was inhibited to 48.1% in A549 cells and 58.7% with GSC treatment at 10 μM, respectively. Due to the more potent anti-proliferation effect of GSC in A549 cells, the following mechanism studies would be carried out on this cell line. In addition, we also selected BEAS-2B cells, a normal human bronchial epithelial cell, for treatment of GSC. As shown in **Figure 2C**, no obvious toxicity of GSC was observed. Taken together, GSC could inhibit the growth of lung cancer cells significantly while showed on obvious toxicity on normal lung cells.

### Effects of GSC on A549 Cell Apoptosis

**FIGURE 3:**
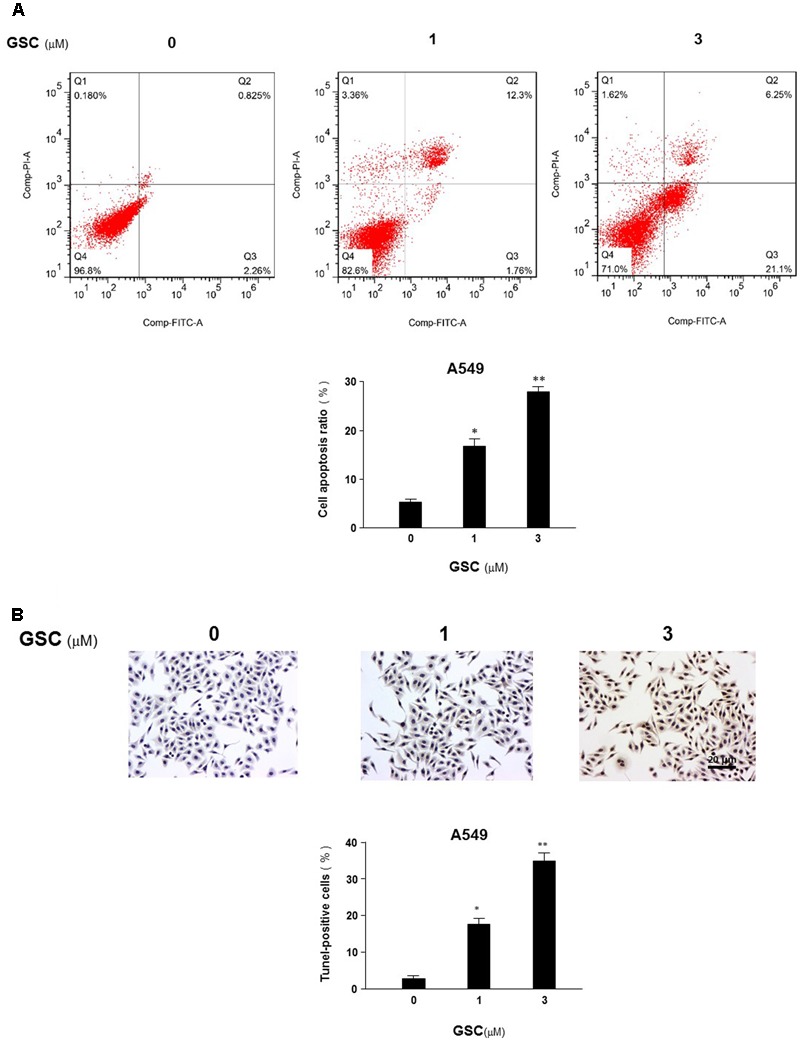
Effects of GSC on apoptosis of A549 cells. **(A)** A549 cells were treated with GSC at different concentrations (0, 1, 3 μM). After 24 h later, all cells were harvested for flow cytometry analysis. Annexin V/PI-stained cells were analyzed with the percentages of apoptosis cells. Each experiments were carried out independently in triplicate. The percentage of annexin V positive cells is also included. **(B)** A549 cells were treated with GSC at different concentrations (0, 1, 3 μM) for 24 h and then TUNEL assays were performed. Apoptosis rate of A549 cells was expressed as the percentage of total cells counted. TUNEL staining profile of A549 cells was also shown. A dark brown DAB signal indicates positive staining while shades of blue–green to greenish tan signifies a non-reactive cell. The experiment results were shown as the mean ± SD of three independent experiments and each was performed in triplicate. ^∗^*p* < 0.05, ^∗∗^*p* < 0.01 (compared with that of non-GSC treated group).

Relationship between GSC on anti-proliferation of A549 cells and apoptosis induction would be revealed by flow cytometry. A549 cells were treated with GSC at 1 and 3 μM, respectively. PI and Annexin V double staining were carried out for apoptosis evaluation. As shown in **Figure 3A**, the number of PI stained cells was increased, indicating apoptosis induction by GSC treatment. The results of TUNEL assays were consistent with the PI staining assay (**Figure 3B**). Compared with untreated cells, 1 and 3 μM GSC induced apoptosis in 18.6 and 35.6% of A549, respectively.

### GSC Induces Apoptosis of A549 Cell in a Caspase-Dependent Manner

**FIGURE 4:**
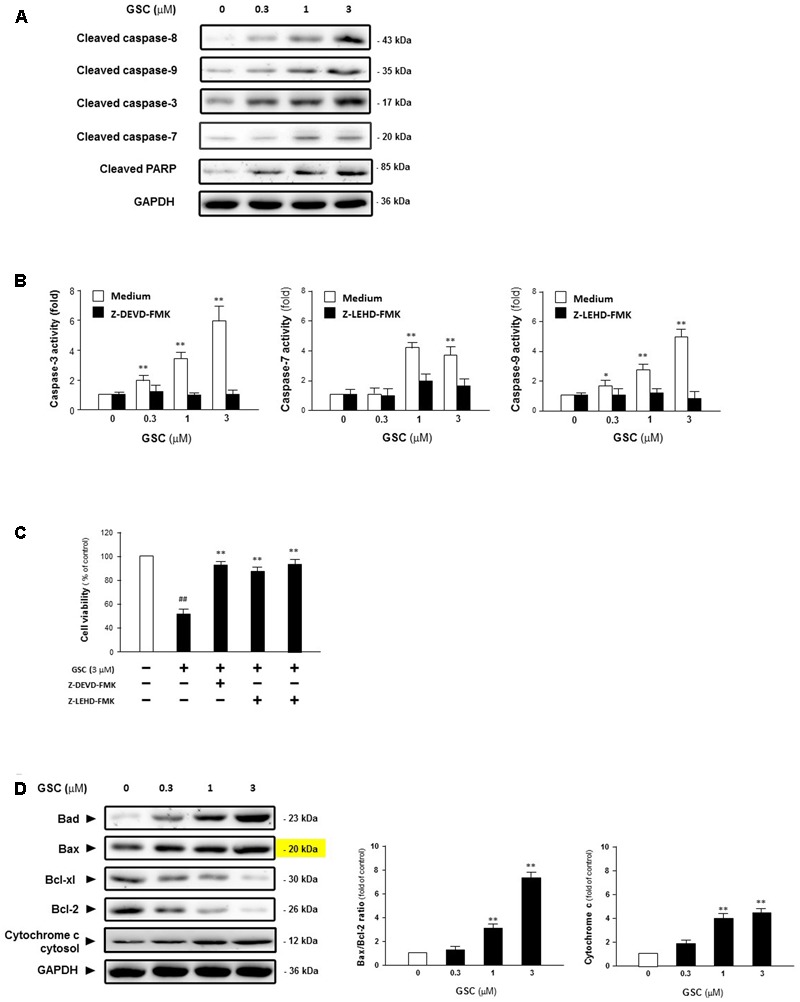
Gleditsia saponin C-induced cell apoptosis is mediated through the caspase-dependent apoptotic pathway in A549 cells. **(A)** Expression levels of Cleaved caspase-8, cleaved caspase-9, cleaved caspase-3, cleaved caspase-7, and cleaved PARP were determined in A549 cells treated with GSC at different concentrations (0, 0.3, 1, 3 μM). **(B)** Activities of caspase-3 and caspase-9 were determined in A549 cells treated with GSC at different concentrations (0, 0.3, 1, 3 μM) for 24 h with or without caspase inhibitors, i.e., z-DEVD-FMK and z-LEVD-FMK. **(C)** Viability of A549 cells were pretreated with caspase inhibitors for 2 h and then co-treated with GSC (3 μM) for 24 h, after which cell viability was determined by MTT assay. **(D)** Expressions of the Bcl-2, Bcl-xl, Bax, Bad Bcl-2 family proteins and cytochrome C were determined in A549 cells treated with GSC at different concentrations (0, 0.3, 1, 3 μM) for 24 h. Then the band intensity was quantified by Image J software. The ratios of Bax:Bcl-2 and cytochrome C were calculated. Data are presented as increased fold of control (GSC at 0 μM) and derived from three different experiments. Data are represented as mean ± SD. ^∗^*p* < 0.05, ^∗∗^*p* < 0.01 (compared with that of single GSC treated group); ^##^*p* < 0.01 (compared with that of non-GSC treated group).

To reveal the mechanism of GSC inducing apoptosis in lung tumor cells, A549 cells were treated with 0, 0.3, 1, or 3 μM GSC for 24 h, and then activation of caspase was determined by Western blot analysis. Shown in **Figure 4A**, GSC treatment increased cleavage of caspase-8, -9, -3, -7 and PARP. The expressions of pro-form of caspase-3, 9, and 8 were not significantly altered by the treatment of GSC (data were not shown). From **Figure 4B**, as compared with the control group, activities of caspase-9, -7, and -3 were increased 5.7-, 3.8-, and 5.9-fold in 3 μM GSC treatment group, With the treatment of two caspase inhibitors z-LEHD-FMK and z-DEVD-FMK, respectively, the GSC induced activation of caspase was abolished, and the cells were prevented from apoptosis (**Figure 4C**). Therefore, GSC might exert apoptotic effects of A549 cells via activation of caspase-mediated apoptotic pathway.

Then, expressions of pro-apoptotic factor, Bad and Bax, anti-apoptotic factors, Bcl-xl and Bcl-2, were determined and the balance between these two groups of factors was examined. As shown in **Figure 4D**, expressions of Bad and Bax were increased while that of Bcl-xl and Bcl-2 were decreased, indicating that GSC treatment activates the intrinsic apoptotic pathway. Besides, the ratio of Bax to Bcl-2 was increased and the release of Cytochrome C was also induced. Based on these data, GSC might induce apoptosis of A549 cells via mitochondrial pathway.

### Effects of GSC on Akt and MAPK Signaling Pathways

**FIGURE 5:**
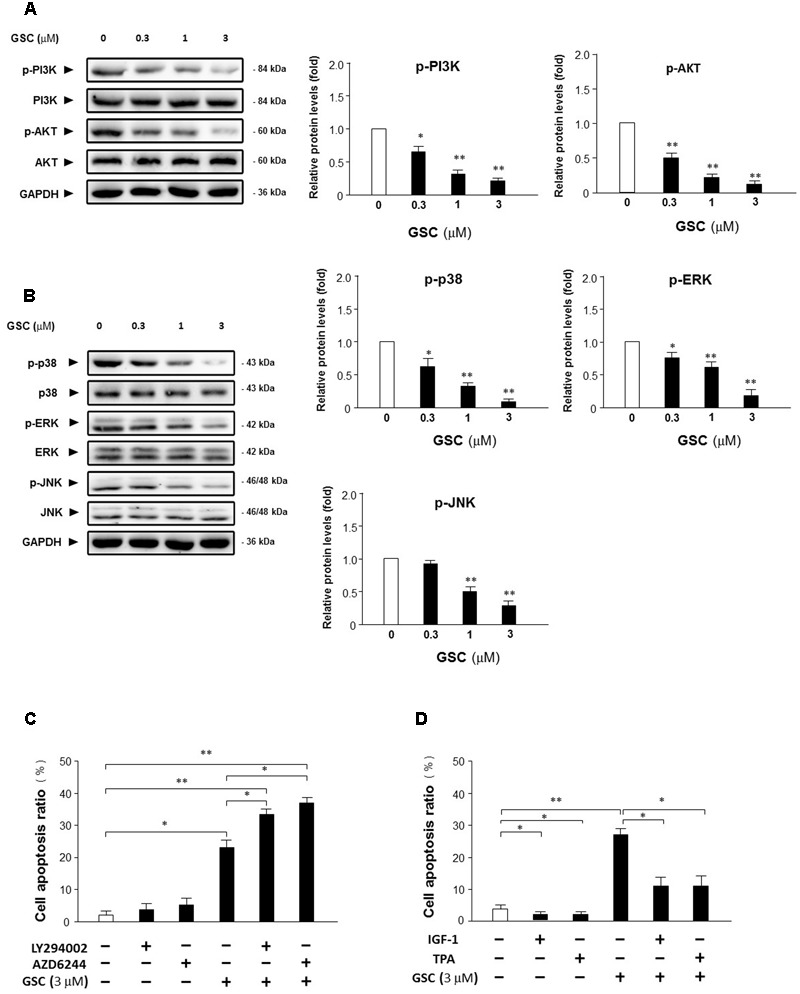
Effects of GSC on PI3K/Akt and MAPKs signaling pathway. **(A)** Expressions of Akt, p-Akt, PI3K, and p-PI3K in A549 cells treated with GSC at different concentrations (0, 0.3, 1, 3 μM) for 24 h were determined with specific antibodies by western blot analysis. GAPDH was used as loading control. Quantifications of the immunoblot data of p-Akt and p-PI3K were also calculated according to band intensity quantified by Image J software. **(B)** Expressions of p-p38, p38, ERK, p-ERK, JNK, and p-JNK in A549 cells treated with GSC at different concentrations (0, 0.3, 1, 3 μM) for 24 h were determined with specific antibodies by western blot analysis. GAPDH was used as loading control. Quantifications of the immunoblot data of p-p38, p-ERK and p-JNK were calculated according to band intensity quantified by Image J software. **(C)** A549 cells were treated with GSC (3 μM), AZD6244 (2 μM), LY294002 (5 μM), the combination of GSC with AZD6244, or the combination of GSC with LY294002 for 24 h before determination of cell death by flow cytometry analysis. Then the apoptosis rate was calculated. **(D)** A549 cells were treated with GSC (3 μM), IGF-1 (10 ng/mL), TPA (10 nM), the combination of GSC with IGF-1, or the combination of GSC with TPA for 24 h before determination of cell death by flow cytometry analysis. Then the apoptosis rate was calculated. Data are representative of three independent experiments. ^∗^*p* < 0.05, ^∗∗^*p* < 0.01.

Afterward, the alterations of critical kinases during survival pathways in A549 cells were further determined to reveal the mechanisms of GSC-induced cell apoptosis. Since MAPK and Akt pathways all play important role in intensifying cell proliferation, inhibiting apoptosis, and potentiating the downstream of NF-κB survival pathway (Karin et al., 2002), whether GSC affecting Akt pathway to induce A549 cell apoptosis was determined firstly. As shown in **Figure 5A**, GSC significantly suppressed phosphorylation of PI3K and Akt on dose-dependent manners. In addition, as shown in **Figure 5B**, GSC also decreased phosphorylation levels of p38 and ERK and JNK dose-dependently. Then, the roles of Akt or ERK in GSC triggering cell apoptosis were evaluated by specific activators and inhibitors. As shown in **Figure 5C**, A549 cells were treated with LY294002 (PI3K/Akt inhibitor) or AZD6244 (MEK inhibitor), which both potentiated GSC-induced apoptosis effect. On the contrary, shown in **Figure 5D**, treatment of IGF-1 (PI3K/Akt activator) or TPA (ERK activator) could attenuate the apoptosis induced by GSC treatment.

### GSC Inhibits Nuclear Translocation of NF-κB p65 and IκBα Degradation

**FIGURE 6:**
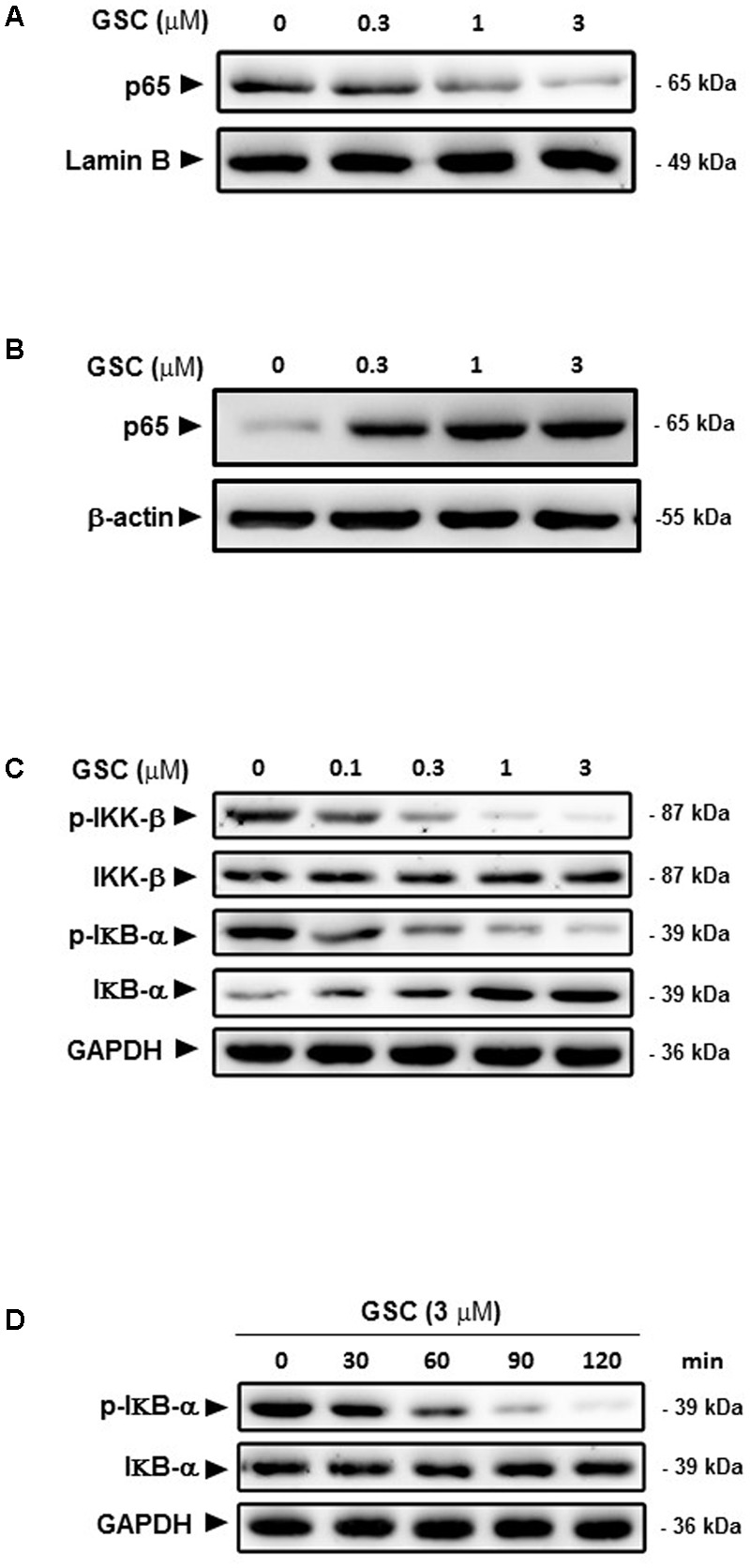
Effects of GSC on the NF-κB signaling pathway in A549 cells. **(A)** A549 cells were treated with GSC at different concentrations (0, 0.3, 1, 3 μM) for 24 h. Expressions of p65 in nucleus were determined by western blotting analysis. Lamin B was used as loading control. **(B)** The treatment of A549 cells with GSC was same as that of **(A)**. Expressions of p65 in cytosol were determined by western blotting analysis. β-actin was used as loading control. **(C)** The treatment of A549 cells with GSC was same as that of **(A)**. Expressions of p-IKK-β, IKK-β, IκBα, and p-IκBα were determined by western blotting analysis. GAPDH was used as loading control. **(D)** The treatment of A549 cells with GSC was same as that of **(A)**. GAPDH was used as loading control. The results shown are representative of three different experiments.

NF-κB, can block apoptosis by upregulation of anti-apoptotic genes such as c-FLIP, Bcl-xl, Bcl-2, and Mcl-1 (Jost and Ruland, 2007). NF-κB inhibition could decrease expression of NF-κB target anti-apoptotic proteins and then promote cellular apoptosis. Based on this scenario, A549 cells treated with series concentrations of GSC might affect the activity of NF-κB. GSC at 0.3 μM did not change the protein expression level of NF-κB/p65 apparently. However, dosages of 1 and 3 μM inhibited the nucleus translocation of NF-κB (**Figures 6A,B**), leading to the inhibition of the transactivation of NF-κB-regulated genes, including Bcl-xl and Bcl-2. Besides, protein expression levels of IκBα were increased after GSC treatment while that of phosphorylated IκBα were decreased (**Figure 6C**). During 2 h, GSC only decreased IκBα phosphorylation levels and showed no obvious change in IκBα levels (**Figure 6D**). All these data suggested that GSC suppressed the release of IκBα from NF-κB. To further elucidate the underlying mechanisms of GSC suppressing NF-κB pathway, the expressions of phosphorylated IKK-β, the upstream regulator of IκBα were determined in GSC treated A549 cells. Displayed in **Figure 6C**, GSC strongly attenuated phosphorylation of IKK-β in A549 cells, which indicated that GSC might inactivate NF-κB signaling pathway by inhibiting IKK-β phosphorylation.

**FIGURE 7:**
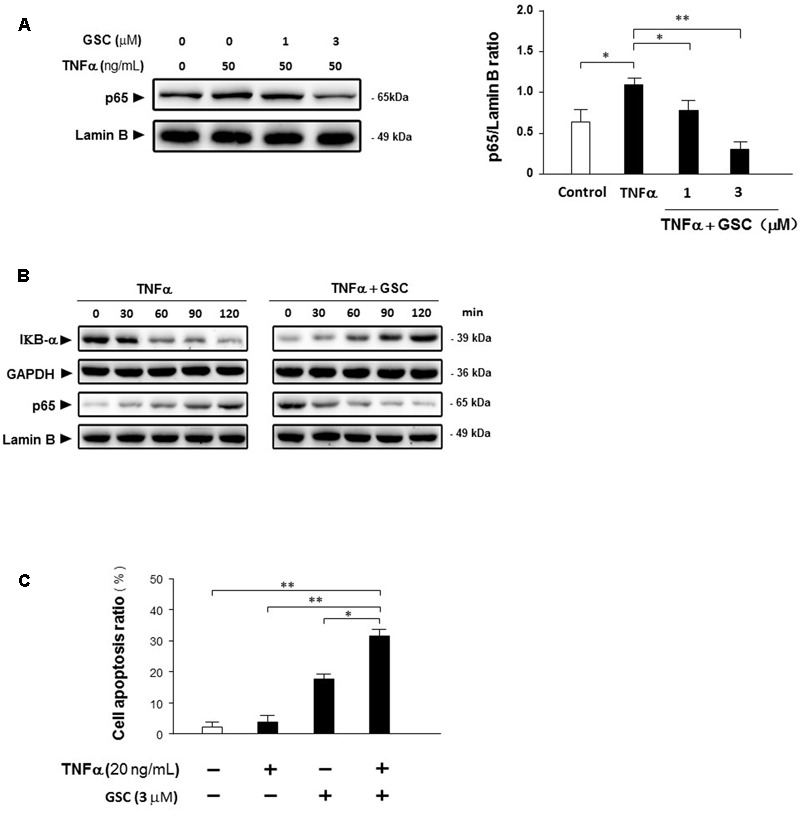
Gleditsia saponin C sensitizes A549 cells to TNFα-induced apoptosis by inhibiting NF-κB activity. **(A)** A549 cells were cultured with 50 ng/mL TNFα in the absence and presence of 1–3 μM GSC for 24 h. Protein p65 were extracted and the expression levels were determined by Western blotting analysis. Lamin B as used as loading control. Band intensities were quantified by Image J software and the results were shown representative of three different experiments. **(B)** A549 cells were cultured with 50 ng/mL TNFα in the absence and presence of 3 μM GSC from 0 to 120 min. Expressions of IκBα and p65 were determined by Western blotting analysis. GAPDH and Lamin B were used as loading control, respectively. **(C)** A549 cells were treated with GSC (3 μM), TNFα (20 ng/mL), and their combination for 24 h. Cell apoptosis ratio was determined by flow cytometry analysis. Data were representative of three independent experiments and represented as mean ± SD. ^∗^*p* < 0.05, ^∗∗^*p* < 0.01 (compared to no treatment group).

It is well-known that inflammation, especially pro-inflammatory stimulation, is also closely related to cancer pathology. Based on previous experimental data, GSC could suppress NF-κB signaling pathway. Whether GSC could suppress NF-κB inducing inflammation under the stimulation of cytokines, the following experiments were carried out. Displayed in **Figures 7A,B**, GSC could inhibit the degradation of IκBα induced by TNFα and thereby blocked NF-κB signaling pathway. Moreover, A549 cells were resistant to TNFα-triggered cell apoptosis (20 ng/mL, **Figure 7C**). Combined with GSC, the synergistic increase in TNFα-triggered cell apoptosis could be discovered.

### *In Vivo* Study of GSC Inhibiting Development of Lung Cancer Xenografts

**FIGURE 8:**
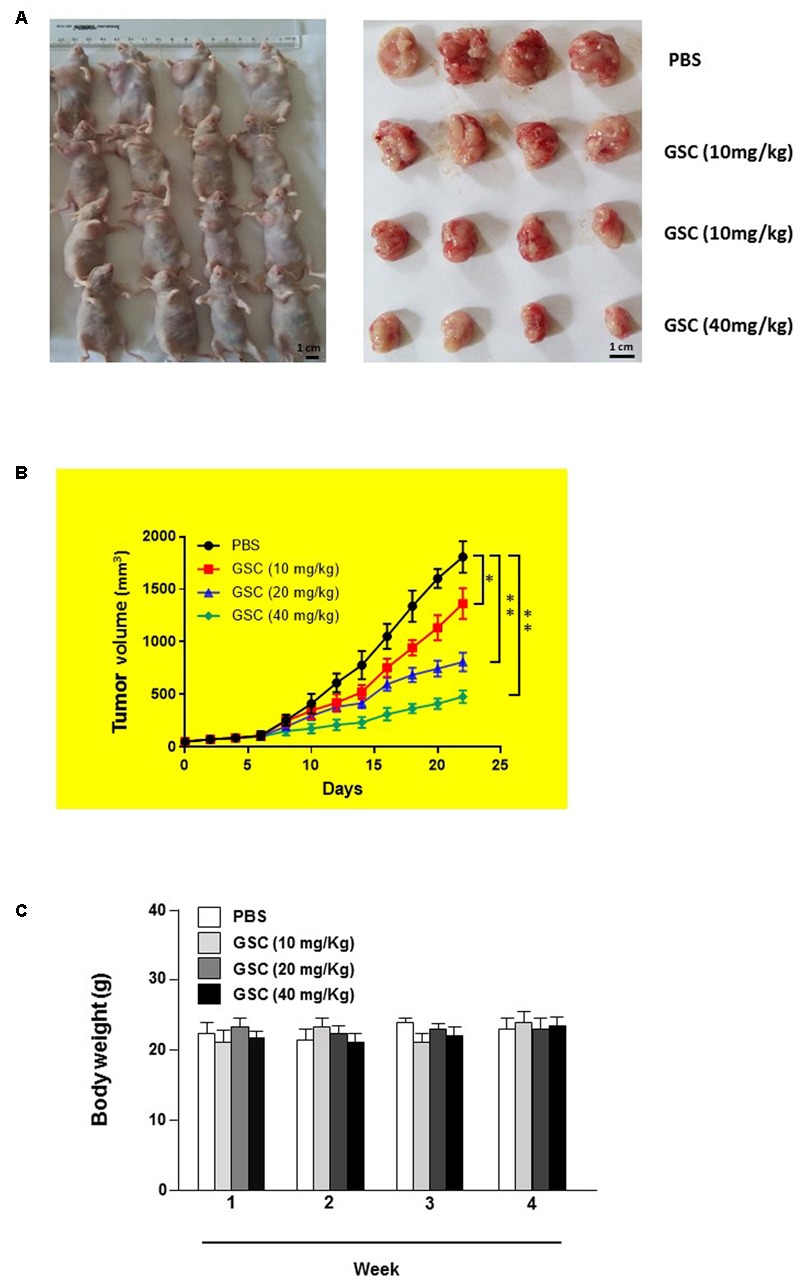
Gleditsia saponin C treatment inhibits *in vivo* tumor xenograft growth in a subcutaneous tumor model in nude mice. **(A)** A549 cells were injected subcutaneously into the dorsal flanks of athymic nude mice. When tumors reached a size of approximately 50 mm^3^, mice were orally treated with GSC at the dosage of 10, 20, 40 mg/kg every 2 day for 21 days. The representative pictures of tumor tissues were also shown. **(B)** At the end of *in vivo* study, the tumors were excised from each group and weighed. The tumor volumes of different treatments were compared. **(C)** The weight of nude mice from each group were determined every weeks. All data were shown as mean ± SD. ^∗^*p* < 0.05, ^∗∗^*p* < 0.01.

The anti-tumor effect of GSC was further evaluated on A549 cell transplanted xenografts tumor model in nude mice. Nude mice were divided into four groups randomly on day 7 post-implantation. Mice were treated orally with series dosages of 10, 20, and 40 mg/kg of GSC every 2 days and the total period was 21 days. Tumors were removed and weighed at the end of the study. Tumor weight was significantly reduced in mice treated with 40 mg/kg GSC, as compared with PBS treated group (**Figure 8A**). Significant tumor suppress of xenograft tumors growth was also discovered in mice receiving treatment with 20 and 40 mg/kg GSC (on days 13–21 vs. control; *p* < 0.01; **Figure 8B**). Meanwhile, however, no obvious change of body weight was found (**Figure 8C**).

**FIGURE 9:**
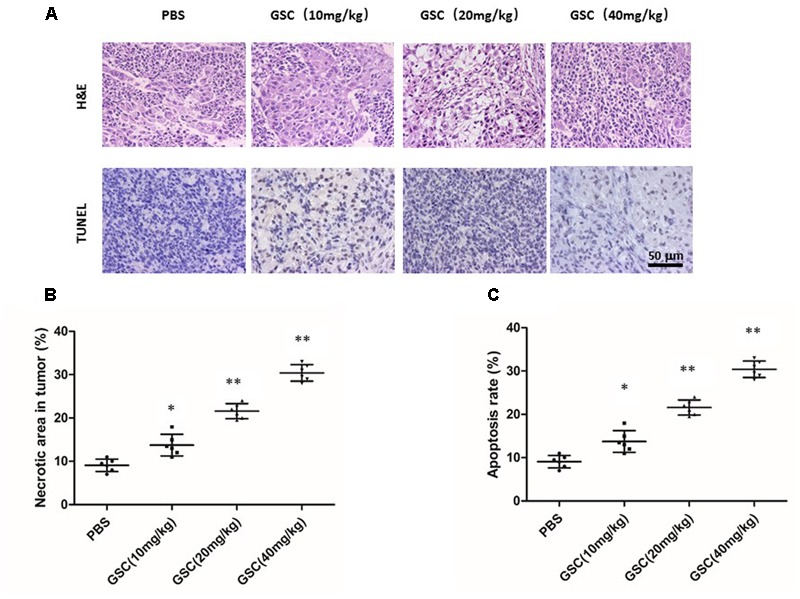
Gleditsia saponin C treatment induces necrosis of tumor and promotes tumor cell apoptosis. **(A)** Necrosis and apoptosis were determined in tumor tissue of nude mice treated with GSC. Tumor necrosis areas were shown by H&E staining and observed under light microscope (×100). TUNEL assay was used to detect apoptotic cells (original magnification, ×200). **(B)** Tumor necrosis was determined by software Image J. Two sections/mouse and three mice were prepared (mean ± SD, ^∗^*p* < 0.05, ^∗∗^*p* < 0.01). **(C)** For apoptosis rate, three fields of the highest density of TUNEL positive cells in each section were counted to determine the percentage of apoptotic cells to total cells. All data were shown as mean ± SD. ^∗^*p* < 0.05, ^∗∗^*p* < 0.01.

Displayed by H&E staining, GSC treated nude mice had more severe necrosis in tumor tissues than those of PBS treatment group (**Figures 9A,B**). The results of TUNEL assays also supported that GSC treatment induced cell death *in vivo* (**Figure 9C**). Taken these data together, GSC induces continuous necrosis within the tumor tissues.

## Discussion

Cancer ranks the third causes of death diseases worldwide. Despite new therapeutics, such as immunotherapy, chemotherapy is still a basic tool in cancer therapies. Induction of tumor cell apoptosis is a primary routine. However, drug resistance is still an intractable question for doctors and patients. Therefore, discovery of compound and development of drugs with more potency and fewer side effects are still challenges for researchers. Discovery of novel chemotherapeutic drugs from natural compounds are promising resources (Bhanot et al., 2011). Saponins are natural compounds well-known for anti-inflammation and anti-tumor (Wang et al., 2009; Jeong et al., 2013; Ma et al., 2017). One of these saponins, GSC is isolated from gleditsiae fructus abnormalis, which possesses the function of eliminating phlegm and commonly prescribed by TCM to treat severe respiratory disorders, most of them are lung cancers. Extracts of gleditsiae fructus abnormalis has been described to possess anti-tumor function both *in vitro* and *in vivo* assays (Chui et al., 2005; Tang et al., 2007). However, the concrete action mechanism has never been explored in details.

On A549 and H1299 cell lines, GSC significantly inhibited cell proliferation and A549 cell line showed robust potency. Based on these phenomena, effects of GSC on regulating signaling pathways accounting for A549 cell survival were explored. Necrosis, apoptosis, and autophagy are there major forms of cell death. Apoptosis is programmed cell death that involves mitochondrial apoptosis pathway, MAPK pathway and so on (Grimaldi et al., 2017; Ugarte-Uribe and García-Sáez, 2017). On mitochondrial apoptosis pathway, our data indicated that GSC induced tumor cell apoptosis by activating caspase-9, -3, led to PARP cleavage and inhibited NF-κB/p65. As to Bcl-2 family proteins, GSC treatment upregulated expressions of Bax and Bad while downregulated expressions of Bcl-2 and then Bax:Bcl-2 ratio was upregulated. According to the roles in regulating permeability of mitochondrial membrane, the Bcl-2 family proteins could be divided into two groups. Bcl-xl and Bcl-2 play anti-apoptotic while Bid, Bak, and Bax play pro-apoptotic roles. Upregulation of Bax:Bcl-2 ratio will result in the release of cytochrome C from the mitochondria and activate mitochondria-dependent caspase cascade to induce apoptotic cell death (Deveraux et al., 2001).

Mitogen-activated protein kinase pathway also plays important roles in cell survival. ERK 1/2 plays an important role in survival while JNK is associated with pro-apoptotic actions (Raman et al., 2007). Our data showed that GSC activated JNK while inhibited PI3K/Akt activation and phosphorylation of p38 and ERK1/2. These data suggested that GSC might induce tumor cell apoptosis by inhibiting MAPK signaling pathways.

Immune regulation plays an important role in cancer pathology. Inflammatory response in presence of NF-κB phosphorylation is frequently observed in malignant tumors and induce cytokine secretion (Shishodia and Aggarwal, 2004; Karin, 2006; Shen and Tergaonkar, 2009). In resting state, NF-κB exists in the cytosol coupled with an inhibitory protein IκB. Once inflammatory stimuli arrives, e.g., LPS and TNFα, IκB is phosphorylated by IKKβ and subsequently degraded, resulting in the release of NF-κB and subsequent nuclear translocation (Häcker and Karin, 2006). TNFα is ligand for necrosis factor receptor to activate the death receptor pathway (Wang et al., 2009). Strategies targeting this signaling pathway have been proposed and investigated for treating cancers. Several reports propose TNFα activates NF-κB and promotes A549 cell survival, angiogenesis, and invasion (Wajant et al., 2003). Therefore, suppression of TNFα-inducing NF-κB signaling can potentiate TNFα inducing cellular apoptosis (Karin, 2006). In current study, we found that GSC attenuated NF-κB activity, inhibited its translocation in nucleus and induced synergistic increase with TNFα of A549 cell apoptosis, which was in line with previous reports that inhibition of NF-κB activation increased TNFα inducing tumor cell apoptosis (Chen et al., 2017). Moreover, GSC reduced TNFα stimulating IκBα degradation and suppressed the nuclear translocation of NF-κB. Taken these data together, GSC might potentiate TNFα inducing apoptosis of tumor cell and reduce the possible inflammatory side effects by NF-κB activity suppression. Due to the potentiality of inducing NF-κB activation and profound inflammatory responses, the clinical usage of TNFα has been limited though it has profound cytotoxic effects on tumor cells (Ashkenazi, 2002). As mentioned before, GSC is a kind of saponin and most of saponins possess the function of anti-inflammation. Therefore, GSC might be applied as a TNFα adjuvant for cancer therapy.

The anti-cancer effect of GSC had also been validated *in vivo* studies. On a tumor xenograft model, GSC also induced apoptosis of cancer cells, and that GSC efficiently inhibited tumor growth on dose-dependent manner. Both of H&E and TUNEL staining showed that GSC effectively inhibited tumor growth attributable to apoptosis. More important, it did not show obvious body weight decline, which indicated fewer toxicity. Combined with *in vitro* antitumor studies, GSC could be developed as a promising drug for cancer therapy. In future, we will further explore the anti-cancer mechanism of GSC based on current foundings. Since PI3K-AKT-mTOR axis and NRLC3 regulation have attracted more and more attention in cancer research, we have preliminarily found that GSC could inhibit mTOR activation in A549 cells and another manuscript is in preparation (Karki et al., 2017a,b). The current research is a preliminary study on the anti-tumor effect of GSC. The bioavailability and other effect of this compound is not very clear and we are still working on these fields. Besides, we will further explore the possible distinct mechanism of GSC on inhibiting tumor growth by genomics, proteomics and metabolomics.

## Conclusion

A natural saponin, abbreviated as GSC, was firstly evaluated as a potent anti-lung cancer compound both *in vitro* and *in vivo* studies. Its concrete anti-tumor mechanism had been revealed from at least four aspects: (1) caspase activation; (2) upregulation of Bax:Bcl-2 ratio; (3) reduction of TNFα inducing NF-κB nuclear translocation; and (4) inhibition of MAPKs and PI3K/Akt pathway. GSC also might ameliorate adverse side effects and drug resistance that occurred in TNFα cancer therapy. Therefore, GSC might be a powerful candidate compound for anti-lung cancer drug development. Though it did not exert obvious toxicity on normal human lung epithelial cells, it cannot be denied that further *in vivo* pharmacological and clinical investigations are required for establishing its promising anti-cancer effect and mechanisms.

## Author Contributions

YC and YH designed the experiments. YC, WH, and YH preformed the molecular biology experiments. YH was in charge of animal studies. YC wrote the main manuscript text. All authors reviewed the manuscript.

## Conflict of Interest Statement

For this research, we do not receive payment or services from a third party. This is a pure research, it might provide information for the cancer drug development company and related readers. No patent and copyright and any pending, issued, licensed and/or receiving royalties are relevant to the work.

## Abbreviations

AIF: apoptosis-inducing factor
ERK: extracellular signal-regulated kinase
GSC: gleditsia saponin C
IκB: the inhibitor of κB
IKK: IκB kinase
JNK: c-Jun N-terminal kinase
LPS: lipopolysaccharide
MAPK: mitogen-activated protein kinase
NSCLC: non-small cell lung cancer
PARP: poly (ADPribose) polymerase
PI: propidium iodide
SCLC: small-cell lung cancer
TNFα: tumor necrosis factor alpha
